# Identification of Gut Microbiome Signatures in Patients With Post-stroke Cognitive Impairment and Affective Disorder

**DOI:** 10.3389/fnagi.2021.706765

**Published:** 2021-08-19

**Authors:** Yinting Huang, Zibin Shen, Wenzhen He

**Affiliations:** Department of Neurology, The First Affiliated Hospital of Shantou University Medical College, Shantou, China

**Keywords:** stroke, 16S ribosomal RNA sequencing, post-stroke cognitive impairment, post-stroke affective disorder, gut microbiota

## Abstract

Stroke (ST), endangering human health due to its high incidence and high mortality, is a global public health problem. There is increasing evidence that there is a link between the gut microbiota (GM) and neuropsychiatric diseases. We aimed to find the GM of ST, post-ST cognitive impairment (PSCI), and post-ST affective disorder (PSTD). GM composition was analyzed, followed by GM identification. Alpha diversity estimation showed microbiota diversity in ST patients. Beta diversity analysis showed that the bacterial community structure segregated differently between different groups. At the genus level, ST patients had a significantly higher proportion of *Enterococcus* and lower content of *Bacteroides, Escherichia-Shigella*, and *Megamonas*. PSCI patients had a significantly higher content of *Enterococcus, Bacteroides*, and *Escherichia-Shigella* and a lower proportion of *Faecalibacterium* compared with patients with ST. Patients with PSTD had a significantly higher content of *Bacteroides* and *Escherichia-Shigella* and lower content of *Enterococcus* and *Faecalibacterium. Parabacteroides* and *Lachnospiraceae* were associated with Montreal cognitive assessment score of ST patients. Our study indicated that the characteristic GM, especially *Bacteroidetes*, could be used as clinical biomarkers of PSCI and PSTD.

## Introduction

Stroke (ST) is the leading cause of death worldwide (Bonita et al., 2004). It can cause some neuropsychiatric diseases, including anxiety, depression, fatigue, apathy, personality changes, mania, and cognitive impairment. It is estimated that 1/3 of ST patients will develop neuropsychiatric disorders shortly after ST (Hackett and Pickles, 2014; Hackett et al., 2014). Post-ST cognitive impairment (PSCI) and post-ST affective disorder (PSTD) are the common complications of ST. The prevalence of depression and cognitive impairment within 3 months after ST are 25–31% and 10–47.3%, respectively (Aström et al., 1993; Jacquin et al., 2014). These two diseases usually coexist in ST patients and have a negative impact on the prognosis of the patient. Cognitive dysfunction is closely related to depression and interacts. Previous studies have shown that the cognitive function of ST patients after antidepressant treatment is normal, and vice versa, which suggest that they may have similar causes. In clinical practice, the limited use of scales and the inability to detect early symptoms have led to the failure of some patients with PSCI to receive correct diagnosis and treatment. Therefore, early evaluation and treatment of PSCI are important to prolong survival time after ST.

Gut microbiota (GM) disorders in neuropsychiatric diseases have been found in some studies (Bains et al., 2019; Nguyen et al., 2019). Recent studies have shown that there are significant differences in fecal microbial diversity in patients with Alzheimer's disease (AD) (Zhuang et al., 2018). Jiang et al. found that the gut microbial structure of patients with active–severe depression has changed, among which, *Bacteroidetes, Proteobacteria, Actinobacteria*, and *Enterobacteriaceae* significantly increased, *Firmicutes* and *Faecalibacterium* decreased significantly (Jiang et al., 2015). The reduced proportion of *Faecalibacterium* leads to chronic low-grade inflammation of the intestinal blood–brain barrier, and is negatively related to the severity of depressive symptoms (Jiang et al., 2015). Recent studies have shown that compared with healthy controls, the composition of the intestinal flora of AD patients has changed (Liu et al., 2019). These reports suggest that the GM may be a crucial regulator of two-way communication between the gut and the brain.

More and more evidence show that intestinal flora can be used as a non-invasive diagnostic biomarker for schizophrenia and type 2 diabetes. It is worth noting that ST patients show obvious intestinal floral imbalance (characterized by a higher abundance of conditional pathogenic bacteria and a lower level of beneficial bacteria). In addition, in patients with PSCI, the abundance of *Fusobacteria* increases and short-chain fatty acids (SCFAs) decrease. However, the composition of the GM in PSCI and PSTD patients has not been evaluated. Therefore, the discovery of the characteristics of the gut microbial composition of PSCI and PSTD patients is of great significance for rehabilitation after ST. Herein, we aimed to investigate the GM composition in ST, PSCI, and PSTD patients. Besides, we also confirmed the characteristic GM of PSCI and PSTD and its potential as a biomarker for the diagnosis of the disease.

## Materials and Methods

### Study Patients

The inclusion criteria for patients were as follows: (1) patients were 40–90 years; (2) patients were ischemic ST; and (3) patients were with infarcts in non-strategic brain regions. Exclusion criteria of patients were as follows: (1) patients with preexisting dementia history and infarct of strategic regions; (2) patients took antibiotics or probiotics (within 3 months); (3) patients with a restrictive diet, gastrointestinal surgery, recent infection, psychosis, severe life-threatening illnesses, communication deficits, and pregnancy. A total of 95 ischemic ST patients were enrolled from the first affiliated hospital of Shantou University, Medical College, which included 19 healthy controls (HC), 27 ST patients, 29 PSCI patients, and 20 PSTD patients. Clinical characteristics of studying subjects are shown in Table 1. The Ethics Committee of the First Affiliated Hospital of Shantou University Medical College approved the study protocol (2019), and all patients gave written informed consent.

**Table 1:** Clinical information of enrolled patients.

**Groups**	**Number**	**Gender**	**Age**	**MOCA score**	**MMSE score**	**Anxiety score**	**Depression scores**	**Hypertension**	**Diabetes**
ST	1	Female	60	27	28	3	3	Yes	No
	2	Female	70	26	28	4	3	No	No
	3	Male	59	26	27	3	0	No	No
	4	Female	61	25	26	5	2	Yes	No
	5	Male	58	30	30	5	4	No	No
	6	Male	64	28	30	0	0	Yes	Yes
	7	Female	67	26	27	2	0	Yes	No
	8	Female	69	26	27	4	4	Yes	No
	9	Female	66	26	28	3	0	No	Yes
	10	Male	51	30	30	0	0	No	No
	11	Male	74	27	27	1	0	Yes	Yes
	12	Male	69	26	28	4	3	Yes	No
	13	Male	54	29	30	6	3	No	No
	14	Female	38	30	30	3	4	No	No
	15	Male	64	30	29	0	0	No	No
	16	Female	55	30	30	5	4	Yes	No
	17	Female	66	27	30	5	5	No	No
	18	Male	62	29	29	2	1	No	No
	19	Female	73	26	29	6	5	Yes	Yes
	20	Female	61	26	28	4	2	No	No
	21	Male	56	30	30	3	3	Yes	No
	22	Male	74	26	26	2	5	Yes	No
	23	Male	68	28	28	3	1	Yes	No
	24	Male	45	30	30	6	2	No	No
	25	Female	74	26	27	0	0	No	No
	26	Male	62	29	29	3	3	No	No
	27	Female	59	29	30	4	4	No	No
	1	Female	62	29	30	9	11	No	No
	2	Male	72	27	28	12	8	No	No
	3	Male	54	30	30	13	14	Yes	Yes
PSTD	4	Female	70	29	30	11	3	No	No
	5	Female	66	29	29	8	8	No	No
	6	Female	74	24	26	11	8	No	No
	7	Male	77	26	28	2	8	No	No
	8	Male	56	29	30	9	8	Yes	No
	9	Female	73	26	26	4	10	Yes	Yes
	10	Female	58	29	30	8	5	Yes	No
	11	Female	69	28	28	8	8	Yes	Yes
	12	Male	64	27	29	10	3	Yes	Yes
	13	Male	54	30	30	8	3	No	No
	14	Female	77	24	25	10	5	Yes	No
	15	Male	47	27	28	7	7	No	No
	16	Female	57	30	30	12	7	Yes	No
	17	Male	61	27	28	12	9	No	No
	18	Male	46	26	27	10	3	No	No
PSCI	19	Male	55	29	30	8	6	Yes	Yes
	20	Male	34	29	29	9	7	No	No
	1	Female	60	0	1	3	3	Yes	Yes
	2	Male	56	0	4	0	2	No	No
	3	Male	59	2	3	1	2	Yes	No
	4	Male	70	12	23	4	2	No	Yes
	5	Male	73	8	17	3	2	Yes	No
	6	Female	62	12	20	4	3	Yes	Yes
	7	Female	65	0	0	0	0	No	No
	8	Male	45	20	22	5	3	Yes	No
	9	Male	74	0	0	3	2	No	No
	10	Male	71	0	0	0	0	No	No
	11	Female	66	0	4	1	1	No	No
	12	Male	80	0	2	2	0	Yes	Yes
	13	Male	74	2	7	5	3	Yes	No
	14	Male	54	0	3	6	4	No	No
	15	Female	55	0	0	0	0	Yes	No
	16	Female	72	5	17	4	2	Yes	Yes
	17	Female	73	0	0	0	0	Yes	Yes
	18	Male	64	21	21	3	2	Yes	No
	19	Female	72	16	18	4	2	Yes	No
	20	Male	70	5	12	3	3	Yes	Yes
	21	Male	58	2	8	0	0	No	No
	22	Male	64	17	20	0	0	No	No
	23	Male	57	2	6	5	3	No	No
	24	Male	57	5	12	3	0	No	Yes
	25	Male	52	10	18	5	2	Yes	No
	26	Male	60	0	1	0	0	Yes	No
	27	Female	47	0	0	0	0	No	No
	28	Male	60	0	4	0	2	Yes	No
	29	Male	50	17	25	3	1	Yes	Yes

*ST, stroke; PSTD, post-stroke affective disorder; PSCI, post-stroke cognitive impairment (PSCI); MOCA, Montreal Cognitive Assessment; MMSE, mini-mental state examination*.

### Sample Collection and Processing

Fresh stool samples from all patients were obtained within 1 week of admission. Stool samples were collected and immediately transferred to the laboratory for repackaging within 15 min. Then 200 mg stool samples were put in a 2-mL sterile centrifuge tubes and labeled. All specimens were processed within 30 min and stored at −80°C. Stool genomic DNA was extracted as described in the previous study (Li et al., 2008; Shkoporov et al., 2018). We put the stool sample in the lysis buffer, added VAHTS DNA cleaning beads, homogenized it in a vortex mixer for 3–5 min, purified with 200 mL of 80% ethanol, and eluted with 24 mL of elution buffer. A 2% agarose gel was used to evaluate the amount of extracted genomic DNA. A NanoDrop spectrophotometer (Thermo Fisher Scientific, USA) was used to determine the DNA purity and concentration. The A260/280 ratio was measured to determine the DNA purity. Then we stored the DNA at −20°C. We amplified the 16S ribosomal RNA gene region of V3–V4 after DNA extraction as described in the previous study (Bu et al., 2018). We performed the high-throughput sequencing on a MiSeq Benchtop Sequencer (Illumina, Singapore, USA).

### Bioinformatics Analysis

Bacterial diversity was determined by alpha diversity and beta diversity. The Wilcoxon rank-sum test was used to identify significant differences in the α-diversity indices between the different groups. Beta diversity was analyzed by using Bray Curtis distances. The beta diversity was visualized *via* principal component analysis, principal coordinates analysis, and non-metric multidimensional analysis. Significant *p*-values associated with microbial clades and functions were identified by linear discriminant analysis effect size (Lefse; Qian et al., 2018). The Kruskal–Wallis test (alpha value of 0.05) and a linear discriminant analysis score >2 were the thresholds of the Lefse analysis. We used Phylogenetic Investigation of Communities by Reconstruction of Unobserved States (PICRUSt) to predict metagenomic functional information according to the operational taxonomic unit (OTU) table (Langille et al., 2013).

## Results

### Alpha Diversity Analysis in ST vs. HC, PSCI vs. ST, and PSTD vs. ST Groups

**Figure 1:**
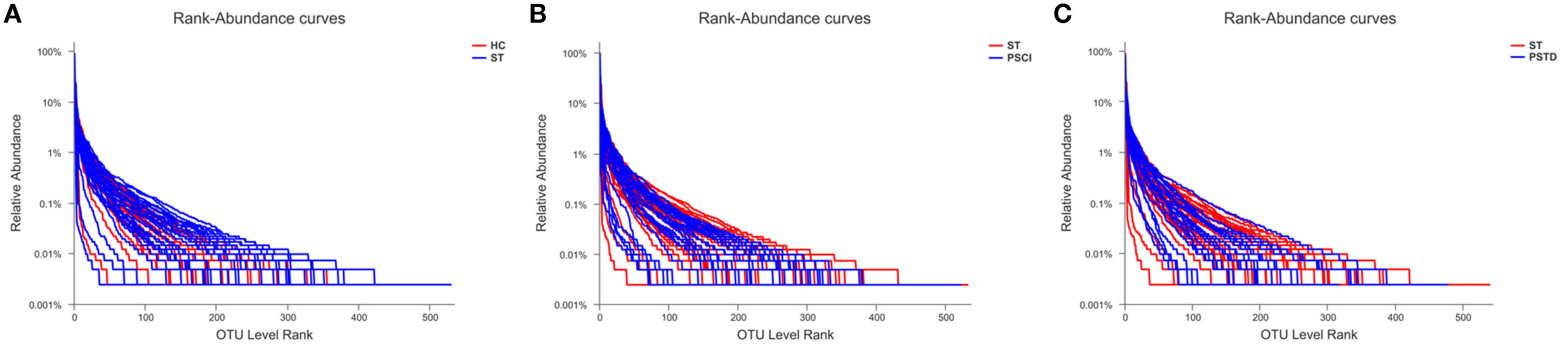
Rank-Abundance curve of the single sample in each group. X-axis and Y-axis means OUT from each sample and the relative abundance of OTUs, respectively. **(A)** ST vs. HC, **(B)** PSCI vs. ST, **(C)** PSTD vs. ST.

**Figure 2:**
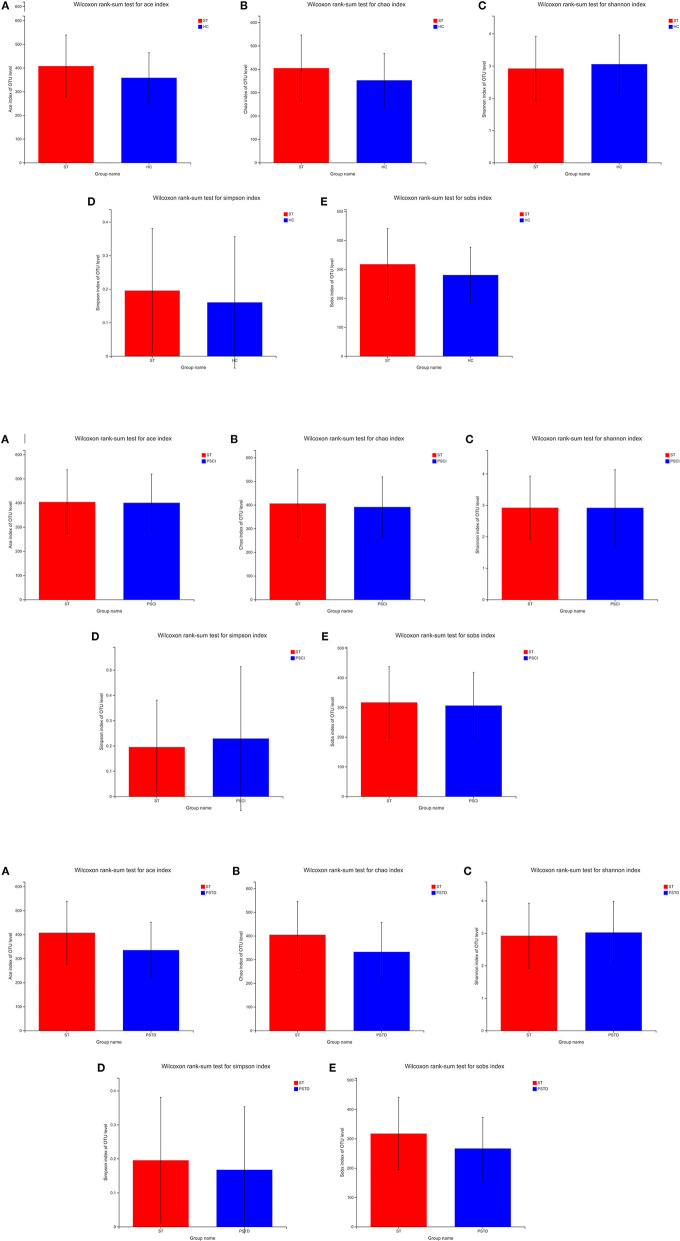
**(A)** Alpha diversity indices in ST vs. HC. **(B)** Alpha diversity indices in PSCI vs. ST. **(C)** Alpha diversity indices in PSTD vs. ST.

**Figure 3:**
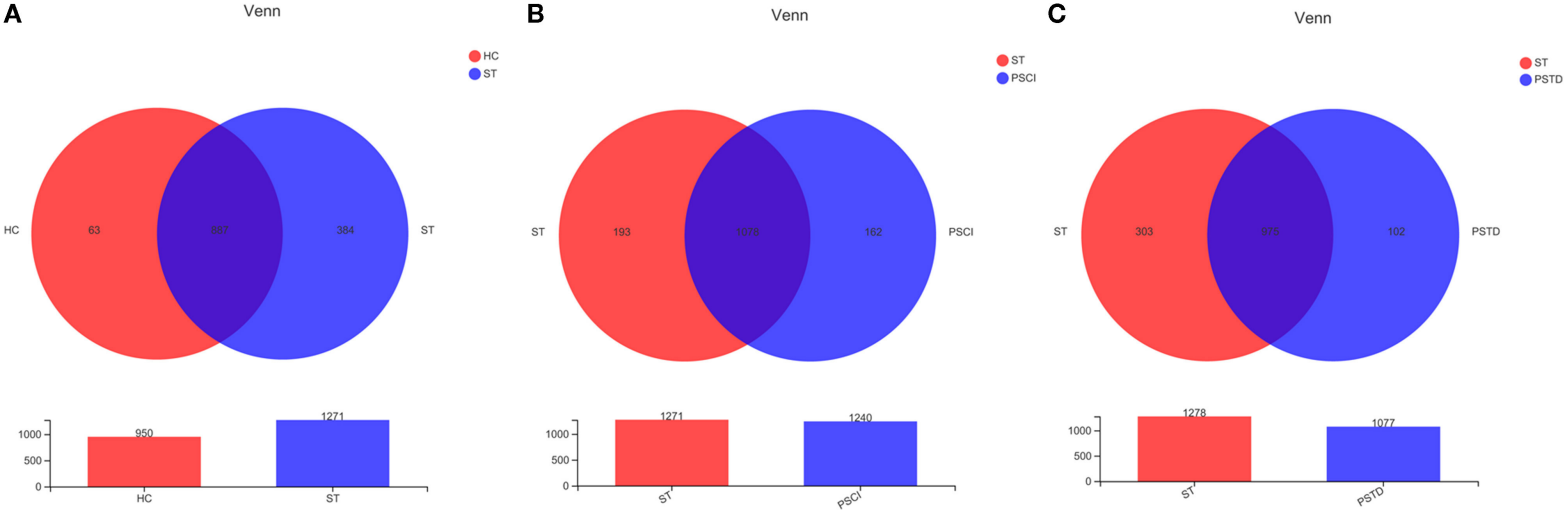
Venn diagrams of ST and HC groups **(A)**, PSCI and ST groups **(B)**, PSTD and ST groups **(C)**.

To evaluate core taxonomic characteristics of ST, PSCI, and PSTD patients, we generated profiles of V3–V4 variable region of the 16s rRNA gene. In Figure 1, rank abundance distribution curves suggested increased richness in ST group and decreased richness in PSCI and PSTD groups compared with the ST group. The alpha diversity indices, coverage evenness, and SD values are shown in Figure 2. As seen from the Venn diagram, there were, respectively 887, 975, and 1,078 common OTUs between ST vs. HC, PSTD vs. ST and PSCI vs. ST groups (Figure 3).

### Alterations in the Composition of Stool Microbiota Associated With ST at the Genus Level

**Figure 4:**
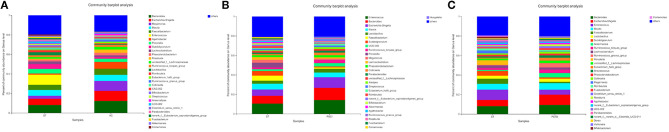
Distribution of the predominant bacteria at genus level of different groups. **(A)** ST vs. HC, **(B)** PSCI vs. ST, **(C)** PSTD vs. ST. The predominant taxa (>1% relative abundance) in each level are shown.

**Figure 5:**
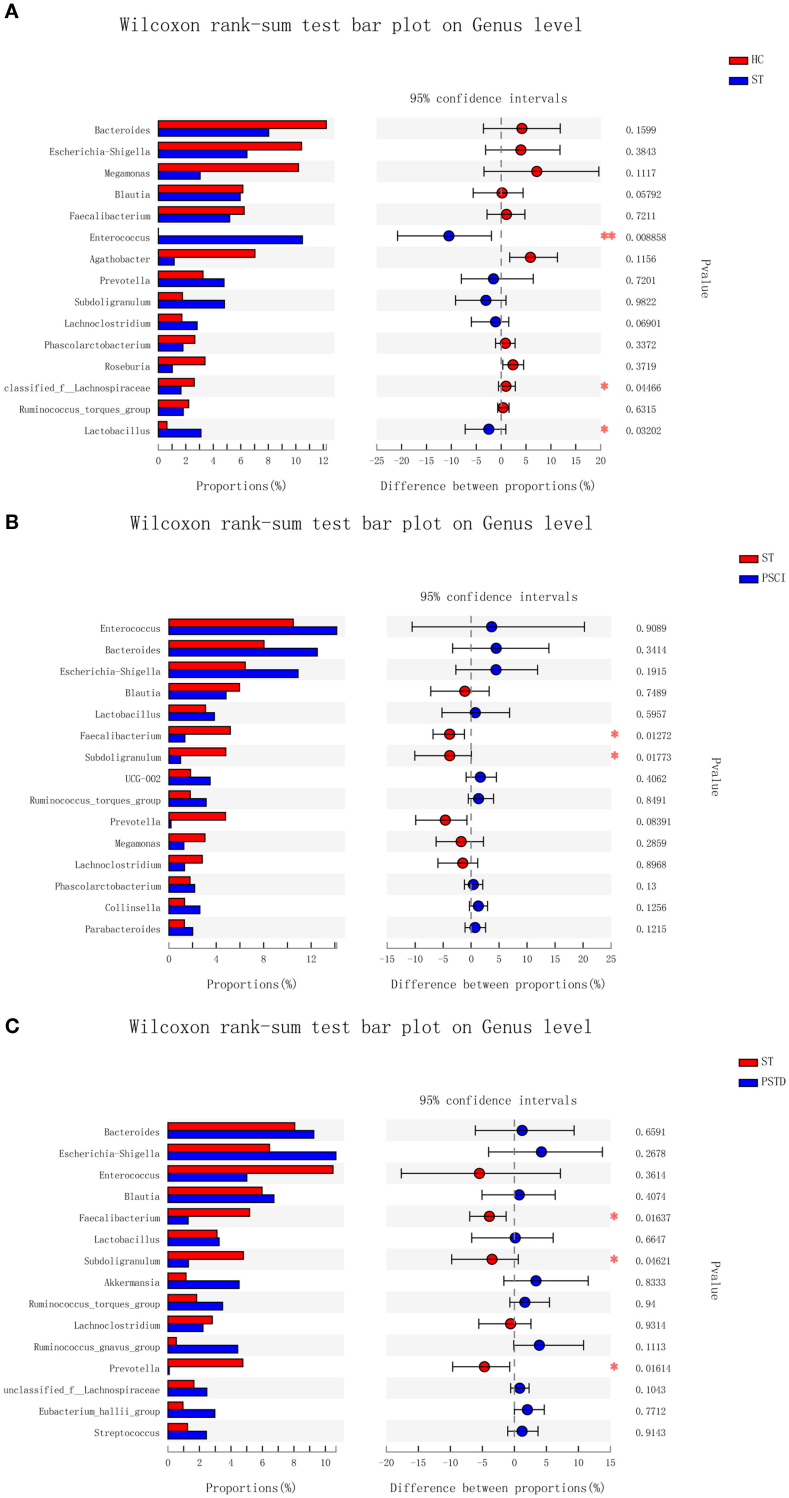
Bar charts of multi-species difference. **(A)** ST vs. HC, **(B)** PSCI vs. ST, **(C)** PSTD vs. ST. ^*^0.01 < *p* < 0.05, ^**^0.001 < *p* < 0.01.

**Figure 6:**
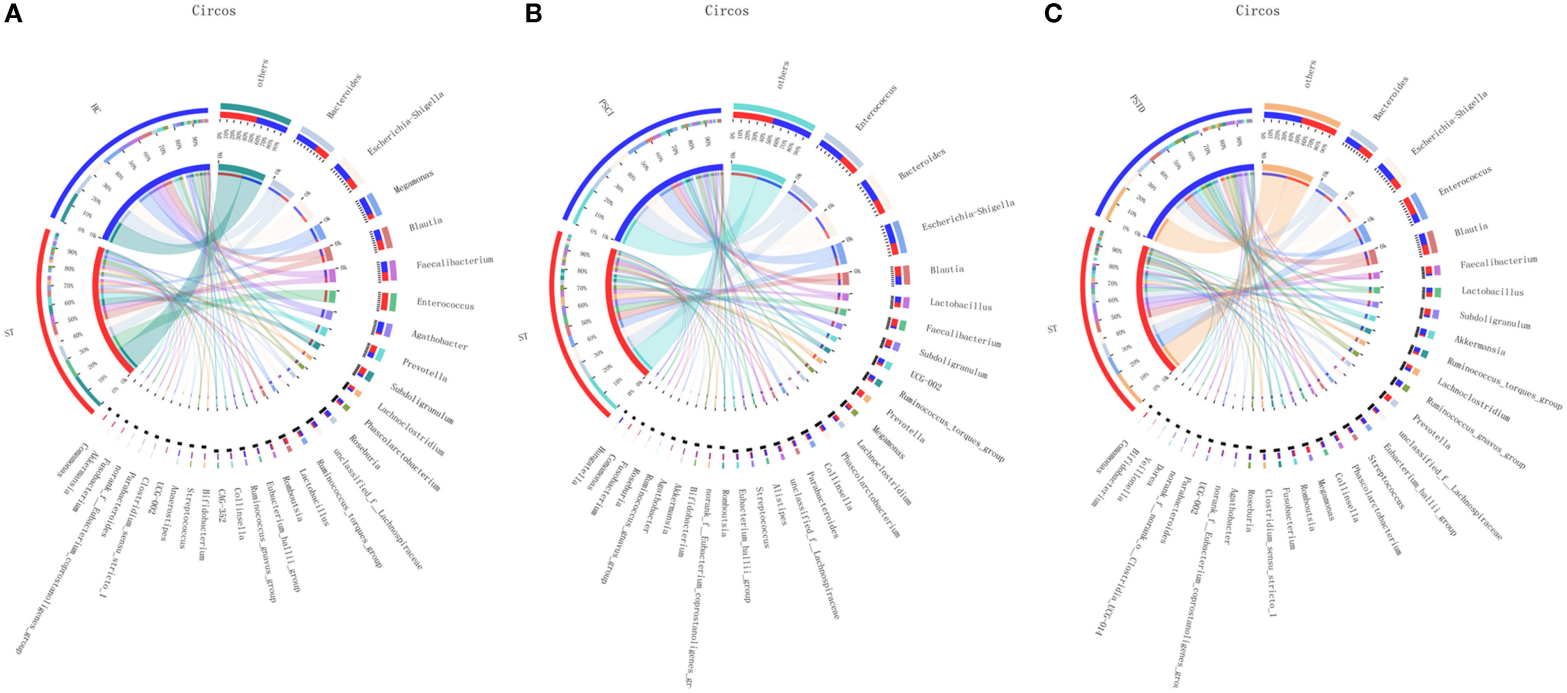
Distribution of the predominant bacteria at genus level in Circos. **(A)** ST vs. HC, **(B)** PSCI vs. ST, **(C)** PSTD vs. ST.

In Figure 4, the relative proportions of dominant taxa at the genus level were assessed. We observed considerable variability in stool microbiota across samples. *Bacteroides, Escherichia-Shigella, Megamonas*, and *Blautia* were the most predominant genera in the ST vs. HC group (Figure 4A). *Enterococcus, Bacteroides, Escherichia-Shigella*, and *Blautia* were the most predominant genera in the PSCI vs. ST group, whereas *Bacteroides, Escherichia-Shigella, Enterococcus*, and *Blautia* were the most predominant genera in PSTD vs. ST group (Figures 4B,C). At the genus level, ST patients had a significantly higher proportion of *Enterococcus* and a lower content of *Bacteroides, Escherichia-Shigella*, and *Megamonas*. By Wilcoxon rank-sum test, there was a significant increase in both the *Enterococcus* (*P* < 0.01) and *Lactobacillus* (*P* < 0.05) genus in the ST patients compared with the HC (Figure 5A). PSCI patients had a significantly higher proportion of *Enterococcus, Bacteroides*, and *Escherichia-Shigella* and lower content of *Faecalibacterium* compared with patients with ST. By Wilcoxon rank-sum test, there was a significant decrease in both the *Faecalibacterium* (*P* < 0.05) and *Subdoligranulum* (*P* < 0.05) genus in the PSCI patients compared with the ST patients (Figure 5B). PSTD patients had a significantly higher proportion of *Bacteroides* and *Escherichia-Shigella* and a lower content of *Enterococcus* and *Faecalibacterium*. By Wilcoxon rank-sum test, there was a significant increase in both the *Faecalibacterium* (*P* < 0.05) and *Subdoligranulum* (*P* < 0.05) genus in the PSTD patients compared with the ST patients (Figure 5C). From the Circos diagram, we further validated these results (Figure 6).

### Alterations in the Composition of Stool Microbiota Associated With ST at the Species Level

**Figure 7:**
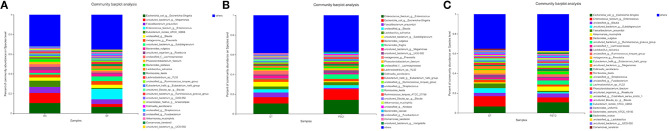
Distribution of the predominant bacteria at the species level of different groups. **(A)** ST vs. HC, **(B)** PSCI vs. ST, **(C)** PSTD vs. ST. The predominant taxa (>1% relative abundance) in each level are shown.

In Figure 7, the relative proportions of dominant taxa at the species level were assessed. We observed considerable variability in stool microbiota across samples. *Escherichia_coli_g__Escherichia-Shigella, uncultured_bacterium_g__Megamonas, Faecalibacterium_prausnitzii*, and *Enterococcus_faecium _g__Enterococcus* were the most predominant species in the ST vs. HC group. *Enterococcus_faecium_g__Enterococcus, Escherichia_coli_g__Escherichia-Shigella, Faecalibacterium_prausnitzii*, and *unclassified_g__Blautia* were the most predominant species in PSCI vs. ST group, whereas *Escherichia_coli_g__Escherichia-Shigella, Enterococcus_faecium_g__Enterococcus, unclassified_g__Blautia*, and *uncultured_bacterium_g__Subdoligranulum* were the most predominant species in PSTD vs. ST group. At the species level, ST patients had a significantly higher proportion of *Enterococcus_faecium_g__Enterococcus* and a lower content of *Escherichia_coli_g__Escherichia-Shigella* and *uncultured_bacterium_g__Megamonas*. PSCI patients had a significantly higher proportion of *Enterococcus_faecium_g__Enterococcus*, and *Escherichia_coli_g__Escherichia-Shigella* and *a* lower content of *Faecalibacterium_prausnitzii* compared with patients with ST. Patients with PSTD had a significantly higher content of *Escherichia_coli_g__Escherichia-Shigella*, a lower content of *Enterococcus_faecium_g__Enterococcus*, and *uncultured_bacterium_g__Subdoligranulum*.

### Characteristics of Beta Diversity Analyses Between ST vs. HC, PSCI vs. ST, and PSTD vs. ST Groups

**Figure 8:**
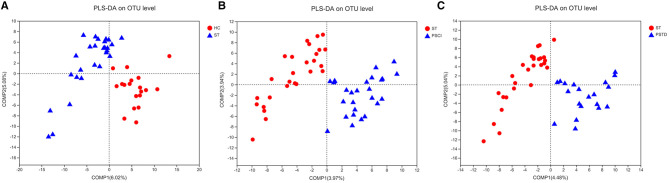
PLS-DA based on OTUs revealed a separation between the **(A)** ST vs. HC, **(B)** PSCI vs. ST and **(C)** PSTD vs. ST groups in the first two principal component score.

In Figure 8, beta diversity analysis was calculated based on partial least squares discriminant analysis (PLS-DA). PLS-DA, based on OTUs, showed a separation between the ST vs. HC, PSCI vs. ST, and PSTD vs. ST groups in the first two principal component scores, which accounted for 6.02 and 5.08%, 3.97 and 3.94%, 4.48 and 5.04% of the total variations, respectively. This indicated that ST may be a key factor that accounts for the changes in the structure of the stool microbiota.

### Correlation Between GM Composition and MOCA Score and Its Subvariables

**Figure 9:**
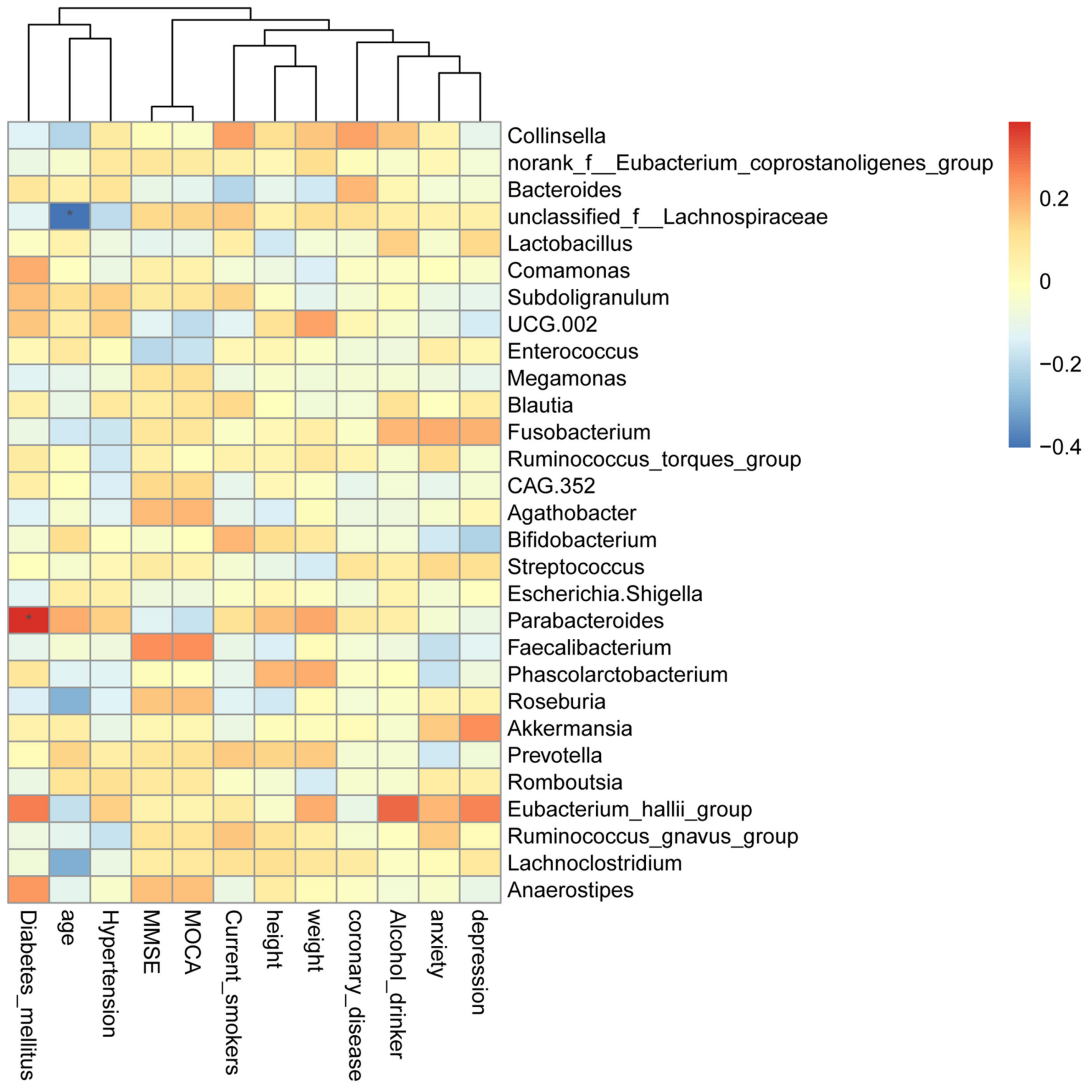
Heatmap of spearman rank correlation analysis between GM and MOCA score and its sub-variables.

The Spearman rank correlation was used to confirm the correlation between Montreal cognitive assessment (MOCA) score and the GM. As shown in Figure 9, *Faecalibacterium, Roseburia, Anaerostipes*, and *Agathobacter* were positively related to the MOCA score. *Parabacteroides, Escherichia-Shigella, Enterococcus, UCG.002, Lactobacillus*, and *Bacteroides* showed a negative correlation. Furthermore, we also investigated the correlation between GM and the MOCA subitems. *Parabacteroides* (P < 0.05)*, Eubacterium_hallii_group* and *Anaerostipes* were found to be positively associated with diabetes_mellitus. *Unclassified_f__Lachnospiraceae, Roseburia*, and *Lachnoclostridium* were negatively correlated with age.

## Discussion

The gut–brain axis (GBA) is a two-way communication network between the brain and the gastrointestinal tract (Tan et al., 2020). GBA is regulated by the central nervous system, autonomic nervous system, enteric nervous system, and hypothalamic–pituitary–adrenal axis (Carabotti et al., 2015). Acute ischemic ST induces dysbiosis of the microbiome, and these resultant changes in the GM affect neuroinflammatory processes and ST outcomes (bottom-up signaling). After ST, up to 50% of patients will experience dysphagia, constipation, gastrointestinal bleeding, and fecal incontinence (Harari et al., 2004; Schaller et al., 2006; Camara-Lemarroy et al., 2014). Gastrointestinal complications after ST lead to the delayed outcome, increased mortality, and progressive neurological dysfunction. It is shown that impaired intestinal flora could also be a risk factor for ST, which affects the prognosis after ST (Li et al., 2019; Zeng et al., 2019).

Differences in microbiota composition are found between models and controls after ST. There are significant differences in the microbiota in all parts of the gastrointestinal tract, even at the phylum level. Growth of *Bacteroides* phylum after ischemia was confirmed in monkeys (Chen et al., 2019). The abundance of *Bacteroides* phylum also increased 3 days after the ischemic ST in mice, which is regarded as a feature of post-ST disorders (Singh and Roth, 2016). In contrast, a clinical study of stool samples collected 2 days after admission showed that patients with acute ischemic ST had decreased *Bacteroidetes* portal protein levels (Yin et al., 2015). In the study of monkeys after focal cerebral ischemia, the relative abundance of *Prevotella* increased, indicating that this type may be related to the inflammatory response after ST. Herein, we detect the GM composition of ST, PSCI, and PSTD patients. Although the bacterial diversity of GM in PSCI and PSTD patients was similar to that of ST patients, the microbial composition was distinct. At the genus level, ST patients had a significantly higher proportion of *Enterococcus* and a lower content of *Bacteroides, Escherichia-Shigella*, and *Megamonas*. PSCI patients had a significantly higher proportion of *Enterococcus, Bacteroides*, and *Escherichia-Shigella* and a lower content of *Faecalibacterium* compared with patients with ST. PSTD patients had a significantly higher proportion of *Bacteroides* and *Escherichia-Shigella* and a lower content of *Enterococcus* and *Faecalibacterium*.

Among the population at high risk of ST, the microbiota alpha diversity index did not change significantly. However, conditional pathogens were found to be enriched in people at high risk of ST, and the abundance of butyrate-producing bacteria was low (Zeng et al., 2019). *Faecalibacterium* is considered to be the main source of butyrate (Machiels et al., 2014). Butyric acid is a SCFA, which plays an important role in maintaining the integrity of the intestinal barrier (Bourassa et al., 2016; Gophna et al., 2017). It is considered as a therapeutic target for brain dysfunction. Chronic intestinal dysbiosis may also affect the production of SCFAs (Chen et al., 2019). In our results, patients with PSCI and PSTD had a lower content of *Faecalibacterium* compared with patients with ST. We speculated that the reduction of *Faecalibacterium* leads to the reduction of butyrate content, which further damages the intestinal barrier and produces proinflammatory cytokines, thus aggravating the disease progression.

Cerebral ischemic ST also causes GM dysbiosis. *Bacteroides* plays a crucial role in the health of the host and can trigger endogenous infections or colitis when the normal microecological balance of the host is impaired (Wexler, 2007). *Escherichia_Shigella* can produce strong endotoxins, increase the intestinal permeability, and cause endotoxemia. *Enterococcus* is an important pathogen of nosocomial and postoperative infections, including the urinary tract and pelvic cavity infections (Sáez-Llorens et al., 1991; Watt et al., 2009). Our results showed that PSCI patients had a significantly higher content of *Enterococcus, Bacteroides*, and *Escherichia-Shigella* compared with ST patients. PSTD patients had a significantly higher content of *Bacteroides* and *Escherichia-Shigella* compared with ST patients. These results indicated that these opportunistic pathogens may play crucial roles in the progression of ST into PSCI and PSTD.

In the correlation analysis between MOCA score and the GM, we found that *Parabacteroides* were significantly positively associated with diabetes_mellitus. *Unclassified_f__Lachnospiraceae* was remarkably negatively correlated with age. Increased abundance of *Parabacteroides* is found in patients with ischemic ST compared with healthy individuals (Li et al., 2019). In patients with depression/anxiety, the content of *Parabacteroides* has changed (Roy et al., 1992; Cheung et al., 2019). In addition, the relative abundance of *Parabacteroides* is positively correlated with gastrointestinal tract (GI) symptoms in elderly patients with type 2 diabetes mellitus (Li et al., 2020). *Lachnospiraceae* plays an important role in modulating GI motility (Yano et al., 2015). It is found that *Lachnospiraceae* dynamically changed with age and positively correlated with anxiety and cognition levels (Duan et al., 2019; Tengeler et al., 2020; Liu et al., 2021). The abundance of *Lachnospiraceae* is significantly decreased in the age-matched PSCI patients (Ling et al., 2020). Moreover, *Lachnospiraceae* had a potential diagnostic value for patients with acute ischemic ST (Xiang et al., 2020). This suggested that *Parabacteroides* and *Lachnospiraceae* are associated with MOCA score of ST patients. However, there are limitations to our study. Firstly, the sample size in each group is small. More stool samples of patients are further needed. Secondly, the deeper mechanism research is not investigated. Further, the animal model is needed in the study.

## Data Availability Statement

The datasets presented in this study can be found in online repositories. The names of repository and accession number (PRJNA734105) can be found in the SRA dataset (https://www.ncbi.nlm.nih.gov/bioproject/PRJNA734105).

## Ethics Statement

The studies involving human participants were reviewed and approved by The First Affiliated Hospital of Shantou University Medical College. The patients/participants provided their written informed consent to participate in this study.

## Author Contributions

YH and WH conceived and designed the experiments. YH and ZS performed the experiments and conducted the statistical analyses. All authors contributed to the article and approved the submitted version.

## Conflict of Interest

The authors declare that the research was conducted in the absence of any commercial or financial relationships that could be construed as a potential conflict of interest.

## Publisher's Note

All claims expressed in this article are solely those of the authors and do not necessarily represent those of their affiliated organizations, or those of the publisher, the editors and the reviewers. Any product that may be evaluated in this article, or claim that may be made by its manufacturer, is not guaranteed or endorsed by the publisher.
